# The impact of area deprivation on access to and utilization of health services in the last year of life: A retrospective population-based cohort study

**DOI:** 10.1177/26323524251332302

**Published:** 2025-10-05

**Authors:** Jackie Robinson, Bert van der Werf, Daniel Exeter, Jinfeng Zhao, Merryn Gott

**Affiliations:** 1 School of Nursing, Faculty of Medical Health Sciences, University of Auckland, New Zealand; 2 Epidemiology and Biostatistics, Faculty of Medical Health Sciences, University of Auckland, New Zealand

**Keywords:** palliative care, inequities, deprivation, rurality, service use

## Abstract

**Background:**

The healthcare needs of people living in areas of high deprivation are complicated by the cumulative effect of the sociodemographic factors known to impact on health outcomes, such as income, housing and education. Of note, for people living in more deprived areas, life expectancy is shorter and the onset of chronic disease and multimorbidity occurs much earlier. While the relationship between area deprivation and access to palliative care is becoming more widely researched, the vast majority of studies to date have focused on referrals to specialist palliative care services. This is problematic given the dominant model of generalist–specialist palliative care in high-income countries which assumes that most people will have a level of palliative care need that can be managed by non-specialist palliative care services.

**Objective:**

To identify associations between area deprivation and the use of generalist and specialist palliative care services in the last year of life.

**Design:**

A retrospective population-based cohort study.

**Methods:**

People aged over 18 years who died between January 2015 and December 2020 were identified within one geographical area of Aotearoa New Zealand. Using the National Health Identifier, deaths were matched to generalist and specialist palliative care service data.

**Results:**

A significant association was found between area deprivation and health service use in the last year of life. Of note, people living in rural areas of deprivation were significantly less likely to receive a hospital (*p* = <0.000) or inpatient hospice admission (*p* = <0.000). They were also less likely to have contact with their general practitioner (*p* = 0.007) or experience a specialist outpatient clinic appointment (*p* = 0.001).

**Conclusion:**

This study has revealed inequities in health service use across generalist and specialist palliative care services for people living in areas of deprivation. Of note, findings have highlighted how rurality amplifies inequities in access to appropriate palliative care. Further research is needed to better understand the consequences of these apparent inequities.

## Plain language summary

The impact of area deprivation in palliative care

The healthcare needs of people living in areas of high deprivation are complicated by the cumulative effect of the sociodemographic factors known to impact on health outcomes, such as income, housing and education. Of note, for people living in more deprived areas, life expectancy is shorter and the onset of chronic disease and multimorbidity occurs much earlier. While the relationship between area deprivation and access to palliative care is becoming more widely researched, the vast majority of studies to date have focused on referrals to hospice services. This is problematic given that people require support from all health services in the last year of life, including hospital services and general practitioner support. The aim of this study was to understand the impact of deprivation on the use of health services in the last year of life. We found that people living in rural areas of deprivation were less likely to receive a hospital or inpatient hospice admission compared to people living in major urban areas. They were also less likely to have contact with their general practitioner or experience a specialist outpatient clinic appointment. This study has revealed inequities in health service use across all health services including hospice services in the last year of life for people living in areas of deprivation.

## Background

A lifetime of socioeconomic deprivation, which for some can be characterized by stigmatization, powerlessness, persecution and injustice, is associated with considerably more suffering at the end of life.^1,2^ Consistently, evidence has shown that people living in areas of high socioeconomic deprivation are more likely to die in hospital^3^ rather than at home or in a hospice.^4^ People living in low socioeconomic conditions are more likely to experience higher pain severity^5^ and less likely to receive adequate pain relief, including in the palliative care setting.^6^ A recent critical review of the literature described the impact of socioeconomic deprivation on increased experiences of “total pain,” constrained choice at the end of life and reduced access to specialist palliative care.^1^

The multifaceted and intersectional array of health and social factors underpinning a lifetime of health inequities highlights that palliative care need at the end of life for people living in areas of socioeconomic deprivation is complex.^7^ Complexity of palliative care need is a key indicator for referral to specialist palliative care services.^8^ If referrals to specialist palliative care were equitable and based on complex unmet needs, it would be logical to assume that people living in deprivation would be over-represented in specialist palliative care referrals. However, the existing evidence, whilst conflicting, points to the opposite. For example, a systematic review and meta-analysis showed that people living in the most deprived areas were less likely to receive a referral to specialist palliative care.^14^ In contrast, a recent study in the United Kingdom found no significant association between area deprivation and referral to hospice.^11^ Of note, compounding this potential inequity is the increased likelihood of unplanned acute hospital admissions at the end of life and death in a hospital setting rather than at home.^9,10^

While the relationship between socioeconomic deprivation and access to palliative care is becoming more widely researched, the vast majority of studies to date have focused on referrals to specialist palliative care services.^12^ This is problematic given the dominant model of generalist–specialist palliative care in high-income countries which assumes that most people will have a level of palliative care need that can be managed by their general practitioner (GP).^13^ In addition, there is an expectation that the GP will remain involved even when a referral to specialist palliative care services is made. Moreover, the palliative care discourse promotes palliative care as being integral to all healthcare providers, regardless of the clinical setting.^14^ This includes care provided by specialist hospital services, such as cardiology, respiratory and oncology services. However, evidence regarding the nature, extent and experience of generalist palliative care is limited as it is often subsumed into the general provision of health and social care. Yet, we do know that people who live in areas of deprivation are less satisfied with the health services they receive. For example, whilst studies have shown that people living in areas of high socioeconomic deprivation visit their GP more often, evidence suggests that the care they receive is of lesser quality and they are less likely to be satisfied with the care they do receive.^15^

The majority of studies exploring the impact of socioeconomic deprivation in palliative care are situated in urban settings. Yet, the impact of socioeconomic deprivation in rural or remote areas is particularly complex and requires an understanding of the unique challenges of these communities. Distance to essential services,^16^ a lack of social housing, poor public transport, unemployment and low income create challenges for rural communities.^17^ These factors can impact significantly on how people experience care at the end of life. However, while rural communities experience unique challenges in accessing palliative care,^18^ evidence has shown that people living in rural areas have a strong sense of community and place great importance on home and landscapes.^19^ Yet little is known about how rurality and socioeconomic deprivation together, impacts on access to and utilization of health and palliative care services in the last year of life.

## Aim

To identify associations between socioeconomic area deprivation and rurality on the use of generalist and specialist palliative care services in the last year of life.

## Design

A retrospective population-based cohort study of people aged over 18 years who died with a residential address in one geographical area of Aotearoa New Zealand (NZ).

## Study setting

The study was situated in one region of NZ consisting of a population of 255,102, most (78%, *n* = 199, 751) of whom live in an urban setting (*n* = 199, 751). The region also consists of a smaller population of 21.6% (*n* = 55, 35) living in a geographically large rural and remote area. In terms of ethnicity, 25.6% of the region’s population identify as Māori. This is significantly higher than the national average of 17.1%. In terms of age, 20% of the region’s population are aged over 65 years.

Overall, the region has high levels of area deprivation compared to the national average, with nearly 60,000 people, or 23.5% living in the highest levels of area deprivation. The two strongest drivers of area deprivation in the region are unemployment and low income. High levels of housing deprivation are also prominent in the large rural area and predictably, the rural parts of the region are also in the most access deprived area zones.

## Methods

Attempts to exclude sudden or unexpected death when exploring the role of palliative care can be problematic.^20^ Identifying people within death data which reflects the “type of death” aligned with traditional palliative care by removing those deaths thought to be “sudden” is difficult. Indeed, excluding deaths thought to be “sudden” or “unexpected,” such as those resulting from trauma, suicide or homicide, runs the risk of excluding people who may benefit from support up to death and/or including bereavement.^21^ Various studies have estimated palliative care need in a population to be anywhere between 37% and 96% of all deaths.^22^ Therefore, for this study, we decided to take a pragmatic approach and include all deaths, assuming that most if not all would benefit from some level of palliative care before and after death, including support during the bereavement period.

The generalist–specialist model of palliative care is well-developed in most Western countries.^14^ This approach to care assumes that most people will have needs at the end of life that can be managed by their usual healthcare providers such as hospital staff and those working in general practice and community settings and is defined as generalist palliative care. Whereas specialist palliative care is care for people with more complex needs and is provided by hospice staff who have been specifically trained in palliative medicine. As few as one-third of people will receive specialist palliative care in Aotearoa New Zealand.^23^ By including all health services involved in the provision of palliative care, we will provide a more nuanced understanding about the impact of deprivation and rurality in end-of-life care.

Deprivation was measured using the New Zealand Index of Multiple Deprivation (IMD18).^24^ The advantage of using the IMD18 as a measure of deprivation is that it takes into account the drivers of deprivation in neighbourhoods. IMD18 uses 29 indicators of deprivation categorized into seven domains; employment, income, housing, health, education, crime and access. There are 6181 areas or data zones across NZ that are assigned a rank of deprivation with 1 being least deprived and 10 being most deprived. Data zones were developed to have populations ranging between 500 and 1000 (mean = 761), to ensure each area was small enough to represent “a neighbourhood” while being statistically robust.^25^ The sample sizes of the participants were relatively small, and using either 5 or 10 socioeconomic deprivation categories would have resulted in cells with a very small number of observations. Therefore, the research team agreed that using three categories was the most robust approach – with level 1 being the least deprived and level 3 being the most deprived.

A database was constructed of people aged >18 years who had died with a residential address at the time of death in a region of NZ between January 2015 and December 2020. The database was constructed by business analysts from the District Health Board (DHB) and included demographic data related to the deceased, ICD10 codes and residential address at the time of death. DHBs (currently referred to as Te Whatu Ora following the restructuring of the NZ health system in 2023) are responsible for a population of people within a specific geographical area.

People whose residential address was an aged care facility were removed prior to the analysis. The reason for this was that the location of an aged care facility does not reflect the effects of the deprivation of a neighbourhood. Residential addresses were geocoded to derive geographical location points using ArcGIS software (https://www.esri.com/en-us/home). These location points were then spatially joined to data zones, and Urban Accessibility Indicator shapefiles enabling associations between patients’ locations, their service utilization, area level deprivation and urban accessibility to be calculated. Urban Accessibility was categorized into major urban, peri-urban and rural remote, based on distance to essential services.^26^

ICD10 codes were used to identify primary diagnosis. Due to the limitations of using multiple diagnostic groups, we categorized diagnosis to cancer or non-cancer. This is appropriate given the continued disparities in access to and utilization of palliative care for people with a non-cancer diagnosis, including within NZ.^27,28^

NZ uses a National Health Identifier (NHI) number that is unique to each individual and is allocated at the time of birth or on the first contact with a health service. We linked the NHI with service data from hospices, hospitals and general practice to identify service use in the 12 months prior to death. In each service data set, the number of deaths differed due to the availability of data over the 5-year period (see Table 1). Each service data set was analysed separately.

**Table 1.** Service utilization data sets.

Service data set	Time period (months)	Number of deaths
Hospital admissions	60	5951
Outpatient clinic	69	7062
General practice contacts	36	3603
District nursing contacts	18	1834
Hospice community contacts	60	5951
Hospice inpatient admissions	60	5951

## Analysis

Categorical data for each service variable were summarized as frequencies observed with percentages. Service variables included hospital admissions, outpatient clinic contacts, hospice community contacts, hospice inpatient admissions and general practice contacts. Given there were only 7062 participants in this study, we dichotomized the service utilization variable for inclusion in the analyses. Comparative group analysis using analysis of deviation was used to identify relationships between categorical groups. Groups included age (five groups), ethnicity (European, Māori, Pacific, other), diagnosis (cancer/non-cancer), urban accessibility (urban, peri-urban and rural remote), area deprivation (least deprived, moderately deprived and most deprived) and gender (male/female).

The binary outcome of having a service contact in the last year of life or not (rather than the number of visits) was used because the number of contacts is zero-inflated for all variables and does not follow a predictable distribution pattern. Moreover, the binary outcome follows the binomial distribution, and the assumptions for the analyses hold.

Logistic regression analysis was undertaken to identify predictors of service use in the last year of life. The regression models consisted of independent variables (IMD18 and its domains) that were linked theoretically to the dependent variable (service use) and/or had a *p* value of <0.05 in the comparative group analysis testing. A multivariate linear regression model was used to explore how well sociodemographic factors were able to predict service use and to identify which of these variables was the best predictor of that outcome. Service use was defined as having at least one contact with the service in the year before death. Cases with missing data related to the independent variables required for the analysis were excluded prior to the analysis.

All statistical analyses were performed by an experienced biostatistician (BW) using R version 3.5.3 and the package ImerTest.^29^ The best logistic model was found by evaluating each deprivation measure in combination with Urban Accessibility. The mode with the lowest Akaike Information Criterion was chosen.^30^ Data were presented as yes/no in relation to receiving a service contact in the last year of life. The STROBE guideline^31^ for reporting of observational studies was followed – see Supplemental File.

## Results

Across the region, there were 11,317 deaths of people aged >18 years recorded between January 2015 and December 2020. The level of area deprivation was assessed using residential address at the time of death therefore any deaths that did not have a valid geocoded physical address that fell within the study region were removed (*n* = 827). All deaths linked to a care facility address at the time of death were also removed (*n* = 3428), leaving 7062 deaths included in the final analysis (see Table 2).

**Table 2.** Demographics (*n* = 7062).

Characteristic	Number
Gender	
Female	43.8% (*n* = 3096)
Male	56.1% (*n* = 3966)
Age (years)	
18–59	17.3% (*n* = 1226)
60–69	17.4% (*n* = 1232)
70–79	26.1% (*n* = 1849)
80–89	29.0% (*n* = 2055)
>90	9.9% (*n* = 700)
Ethnicity	
NZ European	72.9% (*n* = 5149)
Māori	24.7% (*n* = 1747)
Pacific	0.84% (*n* = 60)
Other	1.2% (*n* = 89)
Unknown	0.24% (*n* = 17)
Diagnosis	
Cancer	39.6% (*n* = 2797)
Non-cancer	60.3% (*n* = 4265)
Rurality	
Urban	60.8% (*n* = 4299)
Peri-urban	21.2% (*n* = 1498)
Rural remote	17.9% (*n* = 1265)
Deprivation	
Least	16.1% (*n* = 1141)
Moderate	41.9% (*n* = 2960)
Most	41.9% (*n* = 2961)

The largest proportion of deaths by age was >80 years (*n* = 2055, 29%). NZ Europeans accounted for most (*n* = 5149, 72.9%) deaths and in terms of diagnosis, most (*n* = 4265, 60.3%) had a non-cancer diagnosis. Over 80% (*n* = 2879) of people who died were living in areas of moderate to high levels of deprivation. The majority of the population of people who died were living in major urban areas of the region (*n* = 4299, 60.8%).

Logistic regression modelling was performed to assess the impact of different factors underlying area deprivation on service use in the last year of life. Controlling for age, gender, ethnicity diagnosis and area type, the IMD domain “Access” was found to be associated with experiencing a hospital admission (*p* = 0.019) and a specialist outpatient clinic contact (*p* = 0.014). The “Access” domain considers the distance to health clinics, supermarkets, petrol stations and schools. The IMD domain “Income” was significantly associated with the probability of experiencing a hospice inpatient admission (*p* = 0.004) and was also associated with a general practice contact (*p* = 0.000). The “Income” domain includes weekly government benefit payments, as well as income-tested benefits. Finally, the IMD18 (representing overall area deprivation circumstances) was identified as the best theoretical fit for community nursing contacts, although this was not statistically significant (*p* = 0.0818) – see Supplemental File.

Rurality and deprivation were associated with how people used health services in the last year of life. Differences in health service utilization in the last year of life between people living in areas of most deprivation and urban accessibility were identified as described below.

### Hospital admissions

**Figure 1.**
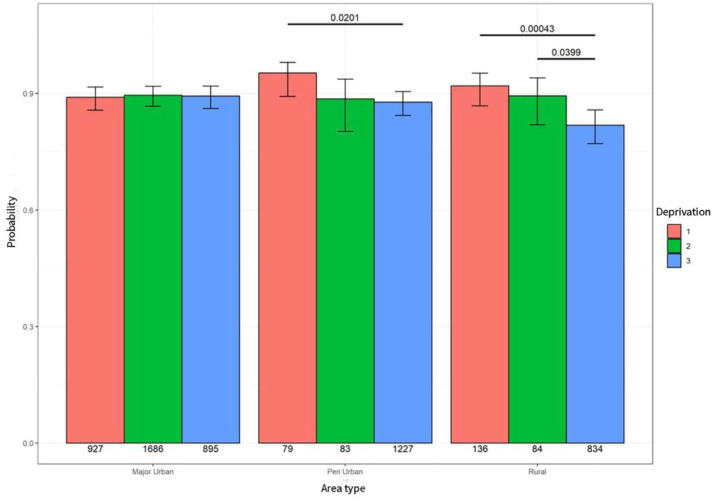
Probability of hospital admission.

In major urban areas, deprivation had little effect on the probability of a hospital admission in the last year of life. Whereas, in peri-urban and rural remote areas, people living in areas of highest deprivation (level 3) were less likely to experience a hospital admission compared to people living in areas of least deprivation (level 1; *p* = 0.021). Furthermore, people living in high area deprivation (level 3) in rural areas were significantly less likely to experience a hospital admission compared to people living in rural areas in least deprivation (level 1; *p* = 0.000) – see Figure 1.

When looking at high deprivation (level 3) across all areas, people living in high-deprivation rural areas were significantly less likely to experience a hospital admission compared to people living in high-deprivation major urban areas (*p* < 0.001) and peri-urban areas (*p* < 0.001) – see Table 3.

**Table 3.** High deprivation and hospital inpatient admissions.

Area type (Dep 3)	*p* Value	Lower 95%	Upper 95%	*p* Value		
				Major urban (Dep 3)	Peri-urban (Dep 3)	Rural remote (Dep 3)
Major urban	0.893	0.861	0.918	1	0.21	<0.001
Peri-urban	0.877	0.843	0.905	0.215	1	<0.001
Rural remote	0.818	0.770	0.857	<0.001	<0.001	1

### Specialist hospital outpatient clinics

**Figure 2.**
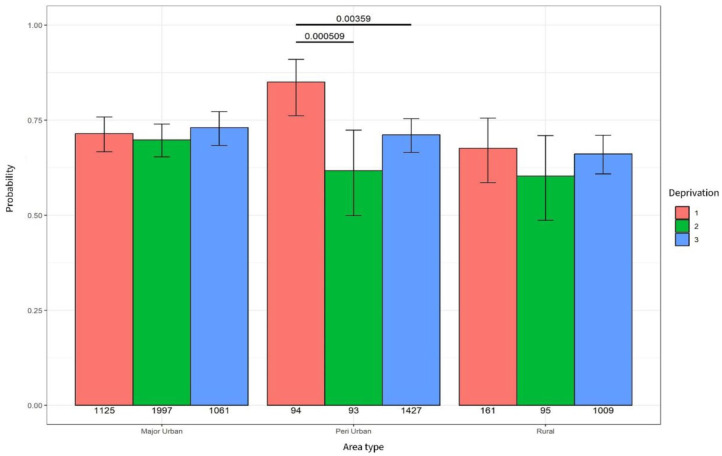
Probability of outpatient contact.

In rural remote and major urban areas, deprivation had little impact on the probability of an outpatient clinic. However, in peri-urban areas, people living in high deprivation (level 3) were significantly less likely to experience an outpatient clinic appointment compared to people living in the least deprivation (level 1; *p* = 0.003) – see Figure 2.

However, when comparing high deprivation (level 3) across different area types (see Table 4), people living in rural remote areas were significantly less likely to experience an outpatient clinic appointment compared to people living in the same level of deprivation in major urban areas (*p* = 0.001).

**Table 4.** Probability of outpatient clinic in high deprivation.

Area type (Dep 3)	*p* Value	Lower 95%	Upper 95%	*p* Value		
				Major urban (Dep 3)	Peri-urban (Dep 3)	Rural remote (Dep 3)
Major urban	0.7303	0.683	0.772	1	0.326	0.001
Peri-urban	0.7114	0.664	0.753	0.326	1	0.014
Rural	0.661	0.608	0.710	0.001	0.014	1

### Community nursing services

**Figure 3.**
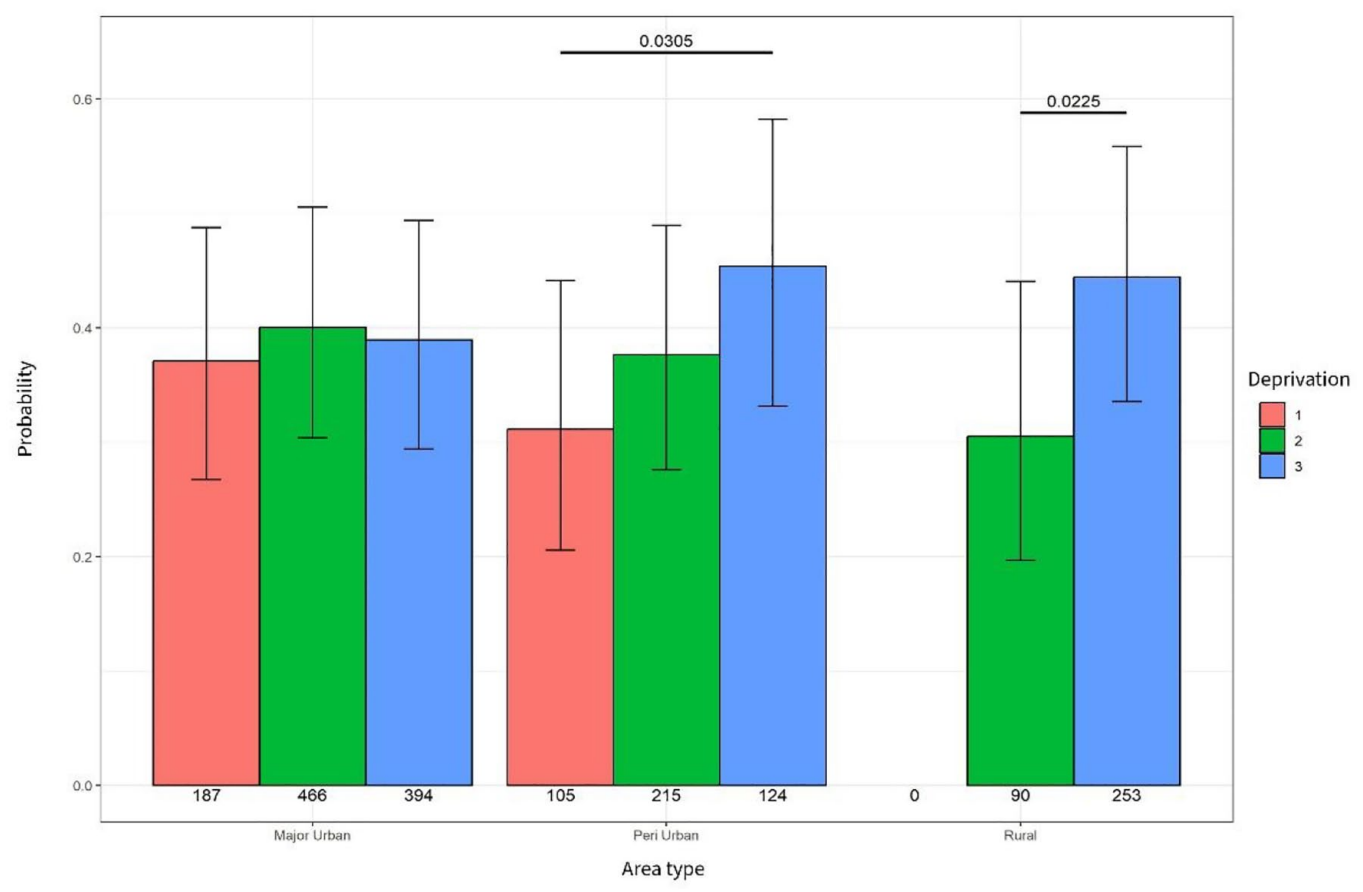
Probability of community nursing contact.

In major urban areas, there was no association between receiving a community nursing contact and living in deprivation. However, in peri-urban areas, a significant association was found between receiving a community nursing service contact and levels of deprivation with people living in high deprivation (level 3) significantly more likely to receive a community nursing contact (*p* = 0.030). Similarly, people living in areas of high deprivation (level 3) in rural and remote areas were more likely to receive community nursing compared to people living in moderately deprived (level 2) rural and remote areas (*p* = 0.02; see Figure 3).

However, when comparing community nursing contacts in people living in high deprivation (see Table 4), there was no statistical difference found between area types (see Table 5).

**Table 5.** Probability of community nursing contact in high deprivation.

Area type (Dep 3)	*p* Value	Lower 95%	Upper 95%	*p* Value		
				Major urban (Dep 3)	Peri-urban (Dep 3)	Rural remote (Dep 3)
Major urban	0.389	0.294	0.493	1	0.213	0.186
Peri-urban	0.454	0.331	0.582	0.213	1	0.859
Rural	0.444	0.335	0.558	0.186	0.859	1

### Hospice services

#### Community hospice nurse contacts

**Figure 4.**
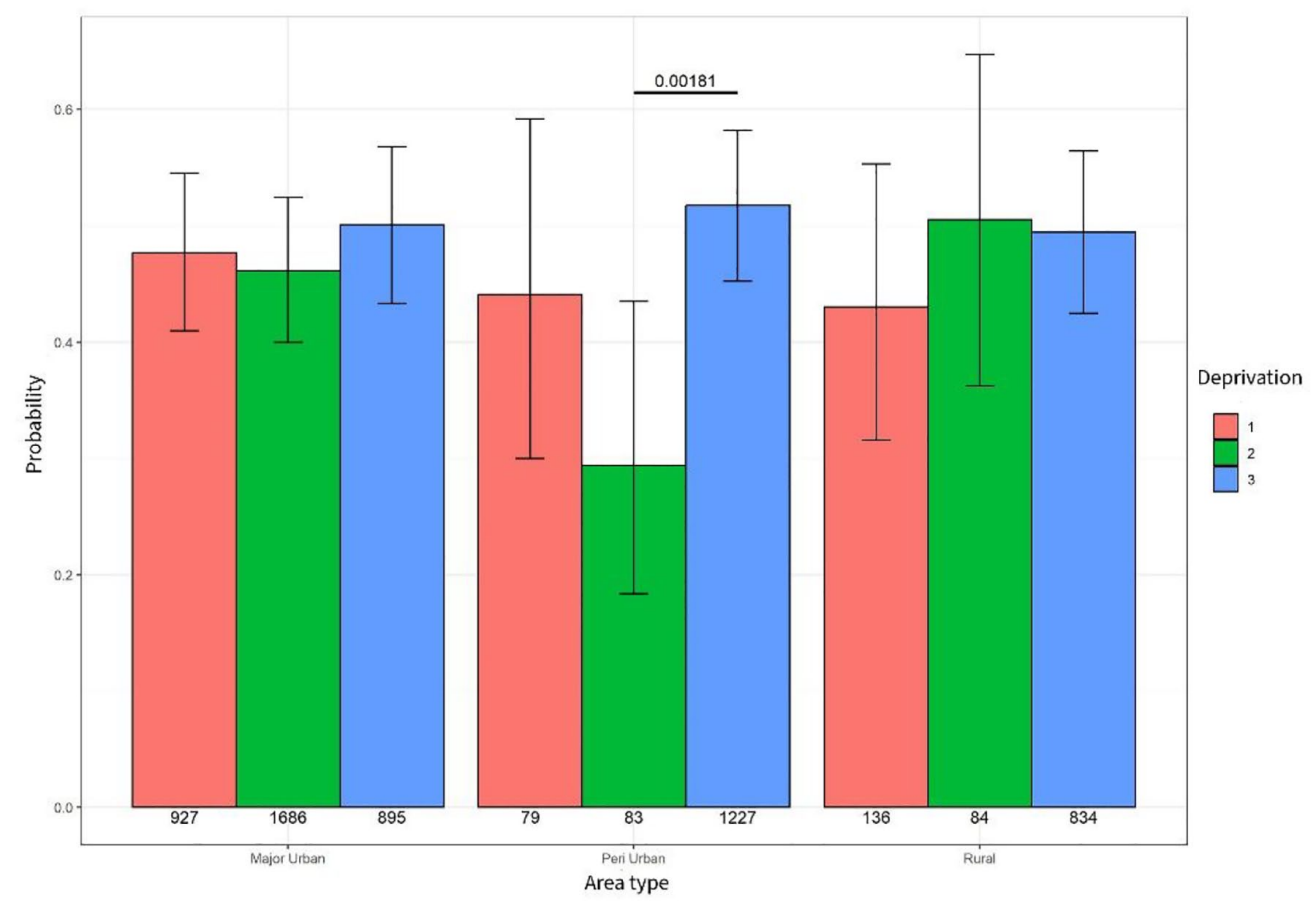
Probability of a community hospice contact.

No association was found between area deprivation and community hospice nurse contacts in major urban or rural areas. However, in peri-urban areas, people living in high deprivation (level 3) were significantly more likely to receive a community hospice nurse contact compared to people living in less deprivation (*p* = 0.001). There was no association found between area deprivation and hospice nurse contacts in rural areas – see Figure 4.

When considering associations between area type and high deprivation, the probability of receiving a hospice community nursing contact was not found to be statistically significant (see Table 6).

**Table 6.** Probability of hospice community nurse contacts in high deprivation.

Area type (Dep 3)	*p* Value	Lower 95%	Upper 95%	*p* Value		
				Major urban (Dep 3)	Peri-urban (Dep 3)	Rural remote (Dep 3)
Major urban	0.389	0.294	0.493	1	0.213	0.186
Peri-urban	0.454	0.331	0.582	0.213	1	0.859
Rural	0.444	0.335	0.558	0.186	0.859	1

#### Hospice inpatient admissions

**Figure 5.**
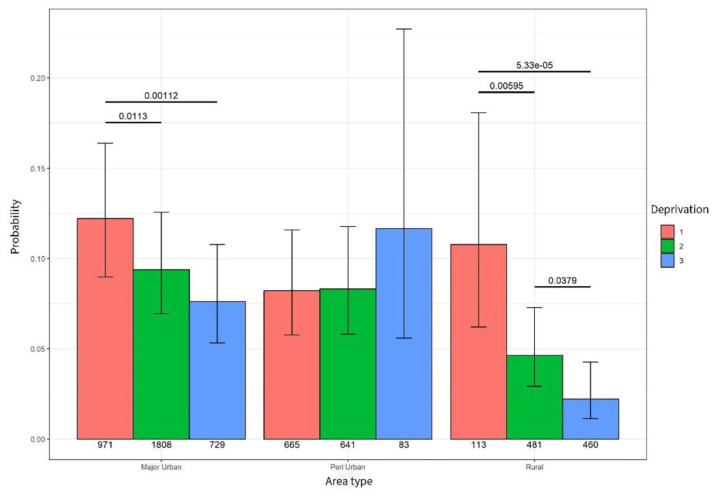
Probability of hospice admission.

An association between hospice inpatient admissions and deprivation was identified in major urban and rural areas. People living in major urban high-deprivation (level 3) areas were less likely to experience a hospice inpatient admission compared to people living in least deprivation (level 1 (*p* = 0.001) and level 2 (*p* = 0.001). Similarly, people living in rural areas of high deprivation were significantly less likely to experience a hospice inpatient unit compared to people living in moderate deprivation (level 2 *p* = 0.003) and people living in least deprivation level 3 (*p* < 0.000) – see Figure 5.

When considering the impact of area type and high deprivation, a statistically significant difference in the probability of a hospice admission was identified. People living in high deprivation (level 3) in rural remote areas were significantly less likely to receive a hospice inpatient unit compared to people living in peri-urban (*p* = 0.000) and major urban areas (*p* = 0.000) – see Table 7.

**Table 7.** Probability of hospice inpatient unit admission in high deprivation.

Area type (Dep 3)	*p* Value	Lower 95%	Upper 95%	*p* Value		
				Major urban (Dep 3)	Peri-urban (Dep 3)	Rural remote (Dep 3)
Major urban	0.076	0.053	0.107	1	0.241	0.000
Peri-urban	0.116	0.056	0.226	0.241	1	0.000
Rural	0.022	0.011	0.042	0.000	0.000	1

### General practice contacts

**Figure 6.**
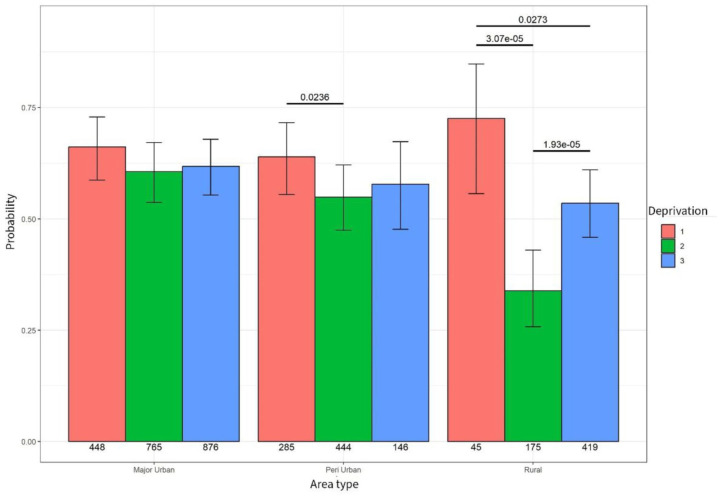
Probability of GP contact. GP, general practitioner.

An association was identified between deprivation and the probability of experiencing a general practice contact in peri-urban and rural areas. In peri-urban areas, people living in moderate (level 2) and high deprivation (level 3) were less likely to see their GP (*p* = 0.0023). In rural areas, people living in high deprivation (level 3) areas were less likely to experience a GP contact compared to people living in moderate deprivation (level 2 *p* < 0.001) and people living in the least areas of deprivation (level 1 *p* = 0.027) – see Figure 6.

When taking into account area type, people living in high deprivation (level 3) in rural areas were significantly less likely to experience a contact with their GP (*p* = 0.007) compared to people living in high deprivation in major urban and peri-urban areas (Table 8).

**Table 8.** Probability of GP contacts in high deprivation.

Area type (Dep 3)	*p* Value	Lower 95%	Upper 95%	*p* Value		
				Major urban (Dep 3)	Peri-urban (Dep 3)	Rural remote (Dep 3)
Major urban	0.618	0.553	0.678	1	0.373	0.007
Peri-urban	0.578	0.476	0.673	0.373	1	0.381
Rural	0.535	0.458	0.610	0.007	0.381	1

GP, general practitioner.

## Discussion

To the best of the author’s knowledge, this is the first study to consider the impact of deprivation and urban accessibility on health service use in the last year of life. By collating data from multiple health services including hospital, hospice, community and general practice, findings from this study have provided a more comprehensive overview of the impact that deprivation and urban accessibility have on service use in palliative care. The findings have highlighted some variability in the association between socioeconomic deprivation, urban accessibility and use of healthcare services in the last year of life. However, a number of key findings are of note.

A key finding from this study was that people living in areas of most deprivation, regardless of geographical location, were less likely to receive inpatient care in the last year of life compared to people living in less deprivation. This includes hospital and inpatient hospice admissions. This was particularly significant for people living in rural remote areas of deprivation. It is well known that in many countries, rural remote areas have experienced a gradual reduction in the availability of essential services.^32^ This has resulted in significant disparities in health outcomes between urban and rural populations.^33,34^ Deprivation compounds the barriers people living in rural and remote communities experience to accessing health services, with limited access to affordable transport and public transport options, poor housing and unemployment.^17^ However, the question remains – does inequitable access to inpatient care in the last year of life contribute to worse outcomes for people with palliative care needs given the contested role of inpatient hospital at the end of life?^7^ There is some evidence that despite the challenges, most people experience some benefits being in hospital and for people living in deprivation the benefits can be significant.^35^ Moreover, admissions to hospital for people with palliative care needs are often presumed to occur as a result of inadequate community-based services.^36^ While findings from this current study suggest that people living in rural and remote areas are more likely to receive community nursing support (hospice and non-hospice nursing services), it could be argued that these services are not a replacement for hospital-based care. Indeed, interventions such as intrathecal analgesia, ureteric and colonic stenting, and the insertion of drainage catheters for the management of malignant ascites and pleural effusions, often require a hospital admission.

Adding to the inequity in hospital admissions for people living in rural areas of deprivation, findings from this study also showed that regardless of geographical location, people living in areas of most deprivation were also less likely to experience a hospice inpatient admission. Hospice inpatient units in Aotearoa New Zealand are available for people with complex palliative care needs. Intensive management of complex physical, emotional, spiritual and psychosocial problems can be addressed in a hospice inpatient unit. Hospice inpatient units can also be an alternative place of care for people where home is not a preferred option.^37^ In Aotearoa New Zealand, approximately one-third of patients referred to a hospice service will have at least one inpatient admission.^23^ In addition, for some people being cared for or dying at home is not an option. People experiencing deprivation may face specific challenges in dying at home relating to the availability of safe, warm, comfortable housing.^1,38^ Furthermore, family or friends may be unable to provide care for financial reasons. Caring for someone at the end of life is known to have a significant financial impact on families.^39^ Indeed, evidence shows that family often accumulate substantial debt and even bankruptcy as they provide direct (transport, food and medication) and indirect costs related to employment, cultural needs and their own health.^40^ For people already living with deprivation, caring for a family member as they near the end of their life can create an added financial burden, particularly if they have to give up paid work to care.^41^ Therefore, one would expect that hospice inpatient services have an important role to play in supporting people with palliative care needs living in deprivation. However, our findings are consistent with those of a recent Danish study which found a statistically significant association between admittance to specialist hospice and hospital palliative care inpatient units and income.^42^ Overall, these findings point to a significant and persisting inequity in access to some aspects of hospice care, namely, specialist inpatient palliative care.^12,43^

Another key finding from this study was that deprivation alone, regardless of geographical location, impacted significantly on the probability of having contact with health services. This is worrying given that the cumulative lifelong effects of neighbourhood deprivation are known to impact on people’s overall health and well-being.^44^ For example, the morbidity and mortality associated with non-malignant life-limiting chronic diseases are higher in deprived populations.^45,46^ A key management strategy for many people living with chronic non-malignant illness such as heart failure and chronic obstructive respiratory disease, is regular contact with specialist cardiology or respiratory services. This is particularly important after an acute exacerbation requiring a hospital admission.^47^ Furthermore, given the responsibility of all clinicians to provide a palliative care approach, contact with specialist clinicians who have some insight into the trajectory of the specific disease and its prognosis is essential.^48,49^ However, findings from this study showed that people living in areas of deprivation were less likely to receive an outpatient clinic contact. This inequity to specialist outpatient consultations in the last year of life was particularly significant for people living in rural remote areas of deprivation, which is likely to be related to the extra time, effort and cost required to access the hospital from these areas.

The central role of the GP in generalist palliative care provision has been well documented in policy^50^ and practice.^51^ However, there is increasing evidence that GP services are not well positioned to provide consistent and reliable care for people with palliative care needs.^52^ This is due to short appointment times, limited home visits and a demanding workload which limits the time GPs have available to provide good palliative care. Our findings provide new evidence that people living in areas of most deprivation are less likely to see a GP in their last year of life when compared to people living in more affluent areas. This was particularly significant for people living in deprivation in rural areas. In Aotearoa New Zealand, there is a partial cost for seeing a GP as well as costs associated with filling a prescription. In addition, similar to other countries, Aotearoa New Zealand has a scarcity of GPs in rural areas. One strategy to address rural inequities in access to GPs is the use of telehealth. However, there may be specific issues for people living in rural areas of most deprivation with difficulties in terms of access to the required equipment, cost and connectivity.^53,54^

This study has revealed inequities in palliative care for people living in areas of most deprivation. These inequities are most marked for people living in areas of deprivation in rural remote communities.

## Limitations

The strengths of this study are that area deprivation and urban accessibility (measured as distance to services and amenities) and its impact on health service utilization across generalist and specialist palliative care services in the last year of life have been considered.

Geographic measures such as area deprivation and urban accessibility provide vital context to analyses, especially when the health datasets used in this study do not obtain any individual-level data from patients regarding their socioeconomic circumstances. Nevertheless, such measures are also liable to biases such as the ecological fallacy, also known as the modifiable area unit problem.^55^ Both the IMD18 and urban accessibility indices provide the average circumstances for a given area, thus areas of relatively high deprivation (tertile 3 in this study) may include a proportion of the population who themselves are not deprived. In remote rural areas, this can be more problematic given that households are more spread out.^56^ Nevertheless, area-based measures are useful for service planning. Data were sourced from multiple different services and in doing so may have impacted the quality of the data received. The risk of this was mitigated through constant reviewing and cleaning of data by one person (JZ) and liaising with data analysts from each service provider. Another limitation is that the data were limited to those with an available residential address at the time of death, excluding people who lived in aged care facilities or who were homeless at the time of death. More work is needed to understand the impact of socioeconomic deprivation on these groups. By including all deaths, there may have been some people whose death was sudden and would therefore have not required palliative care. Some people may have been diagnosed with a life-limiting illness within the 1-year period thereby some contacts with health services may not have been in the context of a palliative care consultation. Finally, limiting diagnosis to cancer and non-cancer limits the generalizability of findings to specific non-malignant disease groups such as people with dementia.

## Conclusion

This study has revealed inequities in health service contacts across generalist and specialist palliative care services for people living in areas of deprivation. Of note, people living in areas of most deprivation in rural areas experience significant inequities in relation to hospital admissions, hospice inpatient admissions, outpatient clinic visits and general practice consultations. These findings have highlighted how rurality amplifies inequities in access to appropriate palliative care. Further research is needed to better understand the consequences of these apparent inequities. Rather than merely viewing them as deficits that need to be corrected, we need to understand the strengths and resilience of rural communities and the contribution this may have to supporting people living in areas of deprivation with palliative care needs.

## Supplemental Material

sj-docx-1-pcr-10.1177_26323524251332302 – Supplemental material for The impact of area deprivation on access to and utilization of health services in the last year of life: A retrospective population-based cohort study Supplemental material, sj-docx-1-pcr-10.1177_26323524251332302 for The impact of area deprivation on access to and utilization of health services in the last year of life: A retrospective population-based cohort study by Jackie Robinson, Bert van der Werf, Daniel Exeter, Jinfeng Zhao and Merryn Gott in Palliative Care and Social Practice

## Statements and Declarations

**Ethical Considerations:** Approval was obtained from the Health and Disability New Zealand Ethics Committee (#20/122). Locality approval was obtained from the region’s Te Whatu Ora. Retrospective data were collected on people who had died.

**Consent to Participate:** Informed consent to participate was waived by the New Zealand Health and Disability Ethics Committee.

**Data Aailability:** The datasets generated during and/or analysed during the current study are available from the corresponding author on reasonable request.
